# 

**Published:** 1912-06

**Authors:** 


### Announcements

Foreign Editors. We expect, beginning with the new volume of the JOURNAL, to add a list of editors for several foreign countries. We have already secured the acceptance of Sir William Osler of Oxford, Eng., and of Professor Dr. P. K. Pel, of Amsterdam, Holland. It is obvious that these and the other gentlemen whom we expect to join our staff, cannot devote their time to routine work on the JOURNAL. They will, however, contribute articles from time to time and will inform our readers of important movements in medical circles in their respective countries.

Original Articles to be Expected. Dr. Ludwig Hektoen of Chicago will publish the lectures delivered in the Harrington course, at the University of Buffalo, in this JOURNAL. The series will probably be divided so as to be printed in the July, August and September issues.

Dr. Louis Faugeres Bishop of New York; The Pulse and its Observation by Nurses.

Dr. George Foy, Dublin; Caesarian Section.

Dr. Anthony Bassler, New York; Medical vs. Surgical Means of Diagnosis and Treatment of Gastro-Intestinal Diseases.

Dr. Thew Wright, Buffalo; Compound Fractures.

Dr. Edith R. Hatch, Buffalo; Acute Pyelitis in Infancy.

